# Multimodal Optical Coherence Tomography for Intraoperative Evaluation of Tumor Margins and Surgical Margins in Breast-Conserving Surgery

**DOI:** 10.17691/stm2022.14.2.03

**Published:** 2022-03-28

**Authors:** D.A. Vorontsov, E.V. Gubarkova, M.A. Sirotkina, A.A. Sovetsky, A.A. Plekhanov, S.S. Kuznetsov, D.A. Davydova, A.Yu. Bogomolova, V.Y. Zaitsev, S.V. Gamayunov, A.Y. Vorontsov, V.A. Sobolevskiy, N.D. Gladkova

**Affiliations:** Oncology Surgeon, Oncology Department; Nizhny Novgorod Regional Oncologic Dispensary, 11/1 Delovaya St., Nizhny Novgorod, 603126, Russia;; Senior Researcher, Scientific Laboratory of Optical Coherence Tomography, Research Institute of Experimental Oncology and Biomedical Technologies; Privolzhsky Research Medical University, 10/1 Minin and Pozharsky Square, Nizhny Novgorod, 603005, Russia;; Director of the Research Institute of Experimental Oncology and Biomedical Technologies; Privolzhsky Research Medical University, 10/1 Minin and Pozharsky Square, Nizhny Novgorod, 603005, Russia;; Junior Researcher, Laboratory of Wave Methods for Studying Structurally Inhomogeneous Media; Federal Research Center Institute of Applied Physics of the Russian Academy of Sciences, 46 Ulyanov St., Nizhny Novgorod, 603950, Russia;; Junior Researcher, Scientific Laboratory of Optical Coherence Tomography, Research Institute of Experimental Oncology and Biomedical Technologies; Privolzhsky Research Medical University, 10/1 Minin and Pozharsky Square, Nizhny Novgorod, 603005, Russia;; Pathologist; Nizhny Novgorod Regional Oncologic Dispensary, 11/1 Delovaya St., Nizhny Novgorod, 603126, Russia;; Head of the Pathological Department; Nizhny Novgorod Regional Oncologic Dispensary, 11/1 Delovaya St., Nizhny Novgorod, 603126, Russia;; Student, Department of Biophysics; National Research Lobachevsky State University of Nizhni Novgorod, 23 Prospekt Gagarina, Nizhny Novgorod, 603950, Russia; Laboratory Assistant, Molecular Biotechnologies Laboratory, Research Institute of Experimental Oncology and Biomedical Technologies; Privolzhsky Research Medical University, 10/1 Minin and Pozharsky Square, Nizhny Novgorod, 603005, Russia;; Head of the Laboratory of Wave Methods for Studying Structurally Inhomogeneous Media; Federal Research Center Institute of Applied Physics of the Russian Academy of Sciences, 46 Ulyanov St., Nizhny Novgorod, 603950, Russia;; Chief Physician; Nizhny Novgorod Regional Oncologic Dispensary, 11/1 Delovaya St., Nizhny Novgorod, 603126, Russia;; Oncology Surgeon, Head of the Oncology Department; Nizhny Novgorod Regional Oncologic Dispensary, 11/1 Delovaya St., Nizhny Novgorod, 603126, Russia;; Professor, Oncologist, Head of the Unit; N.N. Blokhin National Medical Research Center of Oncology of the Ministry of Health of the Russian Federation, 23 Kashirskoye Highway, Moscow, 115478, Russia; Professor, Head of the Scientific Laboratory of Optical Coherence Tomography, Research Institute of Experimental Oncology and Biomedical Technologies; Privolzhsky Research Medical University, 10/1 Minin and Pozharsky Square, Nizhny Novgorod, 603005, Russia;

**Keywords:** breast cancer, breast-conserving surgery, surgical margins, tumor margins, multimodal optical coherence tomography, MM OCT, OCT elastography

## Abstract

**The aim of the study:**

We compare the effectiveness of multimodal optical coherence tomography (MM OCT) in the traditional structural OCT mode and the OCT elastography (OCE) mode in addressing two clinically important tasks: (1) detecting groups of tumor cells at surgical margins during breast-сonserving surgery (BСS) in breast cancer (BC) and (2) identifying breast tumor margins. The obtained results were correlated with corresponding histological sections.

**Materials and Methods:**

The study was performed on 100 surgical margin samples (top, bottom, medial, and lateral — four samples from each patient in total) obtained from 25 patients with BC who underwent BCS (lumpectomy), and on 25 postoperative tumor samples (to determine tumor margins). With MM OCT method, we visually and numerically assessed the scattering (level and depth of OCT signal penetration) and elastic (stiffness values, or Young’s modulus (kPa)) properties of the tumor and non-tumor breast tissue and the obtained values were compared with the results of postoperative histological examination.

**Results:**

In 4 surgical margin samples (out of 100), with the OCE method we identified groups of histologically confirmed tumor cells (“positive” resection margins) at the distance of about 5 mm from the visible tumor margin. The identified zones were larger than 0.5 mm with stiffness of more than 400 kPa in all these cases. However, the structural OCT could not identify these groups of tumors and they were not distinguishable from the surrounding fibrous tissue.

In the areas of tumor into non-tumor tissue transition, structural OCT images detected tumor margins only if they were adjacent to adipose tissue and did not detect them if there were adjacent to non-tumor fibrous tissue. OCE images with high stiffness values (more than 400 kPa) and high contrast showed a clear tumor margin with both adipose and fibrous tissue.

**Conclusion:**

The study demonstarets the potential of MM OCT, particularly its OCE mode, as a real-time method for intraoperative tumor margin and surgical margin assessment in BCS. OCE images compared to structural OCT images visualize higher contrast between different types of breast tissue (adipose tissue, fibrous stroma, hyalinized stroma, tumor cell clusters), as well as more accurate identification of the tumor border and detection of small groups of tumor cells at surgical margins. An algorithm for intraoperative MM OCT examination of the state of the resection margin is proposed in accordance with standard clinical guidelines for achieving clean surgical margins in breast cancer patients.

## Introduction

Breast cancer (BC) remains a topical issue in oncology. In Russia, BC remains the primary cause of morbidity and mortality among malignant neoplasms in women [1]. Early detection is the key to its successful treatment, including breast-conserving surgery (BCS) and non-surgical cancer treatment techniques [2]. The BCS increase in recent years is associated with the improved diagnostic capabilities and early BC detection.

The main criterion for detection and confirmation of the optimal BCS scope is the “negative” surgical margins (areas of the excised breast tissue on the boundary of the surgical incision) [3]. At the same time, scientific studies demonstrated high local BC recurrence (approximately 20%) in patients with tumor cells in the surgical margins during BCS, which requires an urgent intraoperative examination of the surgical margins [4].

At present, there are several techniques for assessing surgical margins, each having certain limitations. For example, in widely used regular microscopic techniques, such as express frozen-section biopsy and cytological examination of cell smears, errors and implementation obstacles are explained by insufficient sampled material, technical difficulties in preparing adipose tissue, need for additional examination time, thus these techniques are of limited efficacy, especially for ductal carcinoma *in situ* [5-8]. Some studies demonstrated the use of intraoperative ultrasound to control surgical margin status; however, due to its low resolution, it has limited sensitivity to small tumor clusters, low reliability for *in situ* cancer imaging [9, 10], and significant dependence on the operator’s skills. A number of optical technologies, such as Raman spectroscopy [11-13] and fluorescence microscopy [14, 15], are used to solve the issue of intraoperative tumor tissue characterization and surgical margin assessment. However, the main limitations of these techniques involve examination of small tissue areas, low penetration depth and scanning speed. Some other techniques are also used for intraoperative assessment of surgical margins [16, 17].

Optical coherence tomography (OCT) is a promising technique for real-time and lable-free intraoperative detection of the breast tumor margins. OCT capabilities are growing with the development of new modalities (polarization-sensitive, elastographic, and microangiographic OCT). In BC studies [18-21], high-resolution OCT was used to classify breast tissue types using a manual OCT guide for *in vivo* identification of tumor cells in both the resection bed and excised samples. The studies demonstrated the possibility of compression OCT elastography (OCE) for BC tissue morphological heterogeneity imaging and intraoperative detection of the tumor margins [22-26]. However, all analysis techniques in these studies are experimental and exploratory in nature, and therefore it is necessary to identify new approaches to OCT examinations similar to real clinical conditions.

At present, there is no consensus on determining the size of the optimal surgical margins. Various authors discuss different margin width ranges — from 1 to 10 mm. The majority (65%) of surgeons consider resection margins of 2 mm or above to be acceptable, although 35% of specialists believe that the width of under 2 mm is acceptable [27, 28]. It is expected that OCT application will allow surgical oncologists to obtain new important information that can improve reliability of optimal resection margins and sensitivity of tumor cells detection at the tumor surgical margin in BCS.

Thus, the relevance of this study is due to objective surgical difficulties related to finding a “negative” surgical margin during lumpectomy and the need for new non-invasive high-resolution techniques of intraoperative real-time imaging.

### The aim of the study

We compare the effectiveness of multimodal optical coherence tomography (MM OCT) in the traditional structural OCT mode and the OCT elastography (OCE) mode in addressing two clinically important tasks: (1) detecting groups of tumor cells at surginal margins during breast-conserving surgery (BCS) in breast cancer (BC) and (2) identifying breast tumor margins. The obtained results were correlated with corresponding histological sections.

## Materials and Methods

### Postoperative patient breast tissue samples

Postoperative samples of tumor and non-tumor breast tissues were obtained from 25 women with T_1–2_N_0_M_0_ (G2–3) stage BC; all of them underwent BCS (lumpectomy) with histological control of surgical margins. The patient age range was 41 to 78 years.

All patients completed a regular set of examinations, including mammography in two projections, ultrasound scan of the mammary glands and neighboring regions, as well as diagnostic punctures followed by cytological examination, when possible.

Patient selection criteria included the following: availability of a nodular formation in the breast tissue, histologically or cytologically verified as a malignant neoplasm, ~10±10 mm in size (5 according to the BI-RADS scale); lack of multifocality and multicentricity signs; lack of dissemination signs.

Depending on the clinical situation and the involved tissue volume, preoperative marking was conducted under ultrasound or mammographic navigation using a 19G marking puncture needle, and then topographic marking was repeated on the skin.

**Figure 1.**
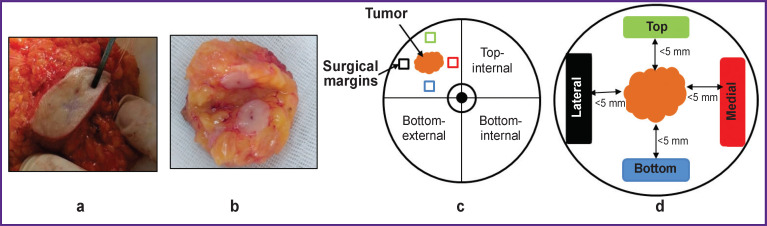
Photo and excision scheme of the examined areas in breast-conserving surgery: (a) a typical preparation after breast-conserving surgery; (b) excision of a part of the tumor node for OCT examination; (c) the examined areas scheme: tumor node and four surgical margins; (d) marking of the surgical margins on the example of a tumor in the top outer quadrant of the right mammary gland

BCS was followed by a planned postoperative histological analysis and additional multimodal OCT examination (MM OCT) *ex vivo* of breast tissue samples from the central part of the tumor node with the neighboring non-tumor tissue, as well as of all marked resection margins of the neighboring non-tumor breast tissue. Breast tissue resection margins were examined at a distance of approximately 5 mm from the visible tumor margin. The removed 4 fragments of the surgical margin were sutured with a surgical thread indicating “top”, “bottom”, “medial”, and “lateral” orientation (Figure 1), similar to the regular histological analysis [2].

With the OCT and OCE images of a breast tissue sample made, the scanning area was marked with histological ink, microscopic sections were prepared from the marked area tissue, and morphological analysis was performed.

Experimental studies on *ex vivo* postoperative breast tissue samples were performed after the receipt of the patients’ informed consent and permission of the Ethics Committee of the Privolzhsky Research Medical University (Nizhny Novgorod, Russia).

### MM OCT device

**Figure 2.**
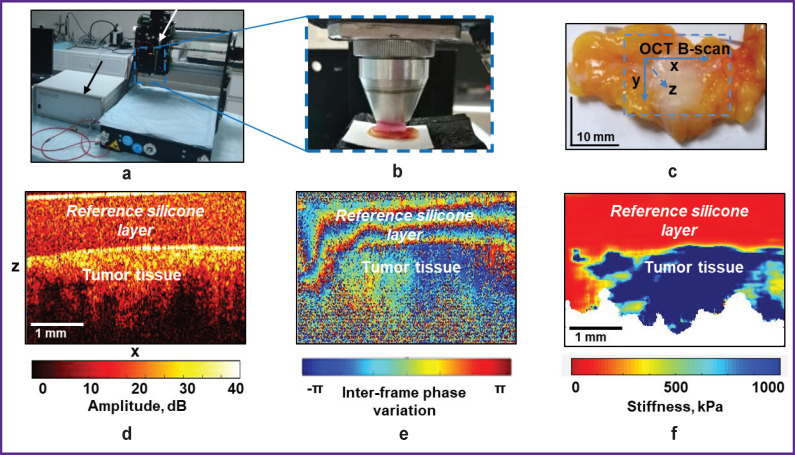
Obtaining and studying structural and elastographic OCT images of breast tissue samples: (a) the MM OCT device (*black arrow*) and device for positioning the OCT probe (*white arrow*); (b) position of the OCT guide on the examined tissue with a reference silicone layer in between; (c) photo of a typical sample of the excised tumor and positioning of the OCT probe during an OCT examination; (d) B-scan of the structural OCT image of the tumor tissue with a silicone, slightly scattering layer on the surface; (e) map of phase difference change between the neighboring B-scans; (f) OCE image of a preselected level of strain in the silicone layer (and thus of a standardized pressure applied to the tissue)

The spectral MM OCT device (Institute of Applied Physics of the Russian Academy of Sciences, Nizhny Novgorod, Russia) with complex real-time acquisition of structural and elastographic OCT images was used (Figure 2 (a)). It uses probing radiation with the central wavelength of 1310 nm, the spectral width of 100 nm, and the focused power of 20 mW. The device depth resolution (in air) is ~15 μm, transverse resolution is ~20 μm, scanning depth in air is ~2 mm, and the rate of spectral reading on the receiving line is 20 kHz/s; recording a 3D image of 2.40×2.40×1.25 mm size takes 26 s [29, 30]. The MM OCT device is equipped with an end fiber optic guide that has an outer objective diameter of 10 mm, which is made to contact the tissue surface under examination (Figure 2 (b)). Precise positioning of the OCT probe on the tissue surface is made using PLRA4 device (Purelogic R&D, Voronezh, Russia) (see Figure 2 (a)), which allows moving the OCT probe along the x–y axes with a minimum step of 10 μm (Figure 2 (c)).

Only the central B-scan of the obtained 3D image (256×256 B-scans) was used for the detailed analysis (Figure 2 (d)) in the study. It is a pseudocolor image in yellow-brown tones where shades of yellow correspond to a high level of the OCT signal and shades of brown — to its low level.

### OСE image analysis

The elastic properties (stiffness) of breast tissue were studied using compression phase-sensitive OCE based on the visualization of deformations created in the tissue by pressing the OCT probe [31-36]. Tissue deformation mapping is based on estimating the interframe variation of the signal phase gradient between the neighboring B-scans (Figure 2 (e)). The greater the variation of the phase gradient of the OCT signal, the higher the percentage of tissue deformation. The technique of OCE compression with a reference silicone layer of certain stiffness (100 kPa in this work) on the biological tissue surface is used to evaluate its elastic properties (Young’s modulus (kPa)) with the refinement of ~30–50 μm (scale ten cells). Simultaneous estimation of strain in the reference silicone layer and tissue during its stress allows identifying its “stress–strain” dependence [35, 36]. Young’s modulus of the examined breast tissue was calculated as the decline in the “stress–strain” nonlinear dependence for the tissue with the selected pressure on the tissue. The quantitative evaluation and comparative analysis of the breast tissue OCE images were carried out using the standardized tissue pressure range (2±1 kPa). Such standardization is important because studies of BC samples showed that the stress/strain ratio of such tissues can manifest pronounced nonlinearity [37, 38]. This means that Young’s modulus can change multi-fold under moderate deformation (several percent).

The B-scans of the OCE images in this study were presented as color-coded maps for Young’s modulus ranging from several kPa to 1000 kPa. The range of stiffness values for elastographic maps was chosen so that the colors best represent different types of tissue (tumor and non-tumor). The authors’ earlier publications [22, 23] define typical ranges of the elastic modulus (Young’s modulus) of five major morphological components of breast tissue by targeted comparison of histological and OCE images. The stiffest areas (blue-green color — over 400 kPa) indicate the presence of tumor cell clusters (scale of at least 300 μm), whereas the softest areas (red color — below 100 kPa) show adipose and unaltered connective tissue. Tissues with transitional stiffness (dominating orange and yellow, which correspond to ~100–400 kPa) contain such degenerative changes in the stroma of the mammary gland as fibrosis or hyalinosis of collagen fibers, as well as lymphohistiocytic inflammation. Thus, Young’s modulus of 400 kPa and above based on visual analysis of OCE images is considered the detection threshold for tumor cell complexes in the present study.

### Histological analysis

After MM OCT imaging, breast tissue samples were fixed in 10% neutral formalin solution for 48 h and then were covered in paraffin. The plane of serial histological sections coincided with the OCT and OCE images plane. To establish a general clinical diagnosis, serial sections were stained with hematoxylin and eosin. Histological specimen were assessed using a Leica DM2500 microscope (Leica Microsystems, Germany) equipped with a DFC 245C digital camera.

The pathologist divided all breast tissue samples into groups according to their morphological structure: adipose tissue (lipomatosis) (n=31); adipose tissue with streaks of connective tissue (n=28); fibrous tissue with dilated ducts/lobules and layers of connective tissue (n=16); sclerosing fibrocystic mastopathy (n=23); ductal carcinoma *in situ* (DCIS) (n=2); invasive ductal cancer (IDC) (n=22) and invasive lobular cancer (ILC) (n=3).

In case of BCS, the main criterion for surgical intervention radicality is the “negative” surgical margins. According to the standard morphological conclusion, a “positive” surgical margin has tumor cells or cancer *in situ* along the surgical margin. In case of a “negative” surgical margin, tumor cells are not found in the margins of the excised breast tissues [39, 40].

125 samples in total were obtained and analyzed by OCT and histologically.

## Results

MM OCT examination of primary tumor samples was used to identify exact breast tumor margins as well as to determine microstructural features of IDC and ILC. Out of 100 surgical margins, 96 specimens were identified

### Detection of the surgical margin status in breast-conserving surgery

OCT and OCE images for visual analysis of the scattering and elastic properties of the breast tissue allowed differentiating the samples as “negative” and “positive” in all cases of studying surgical margins at a distance of approximately 5 mm from the visible tumor margin confirmed by histological examination.

**Figure 3.**
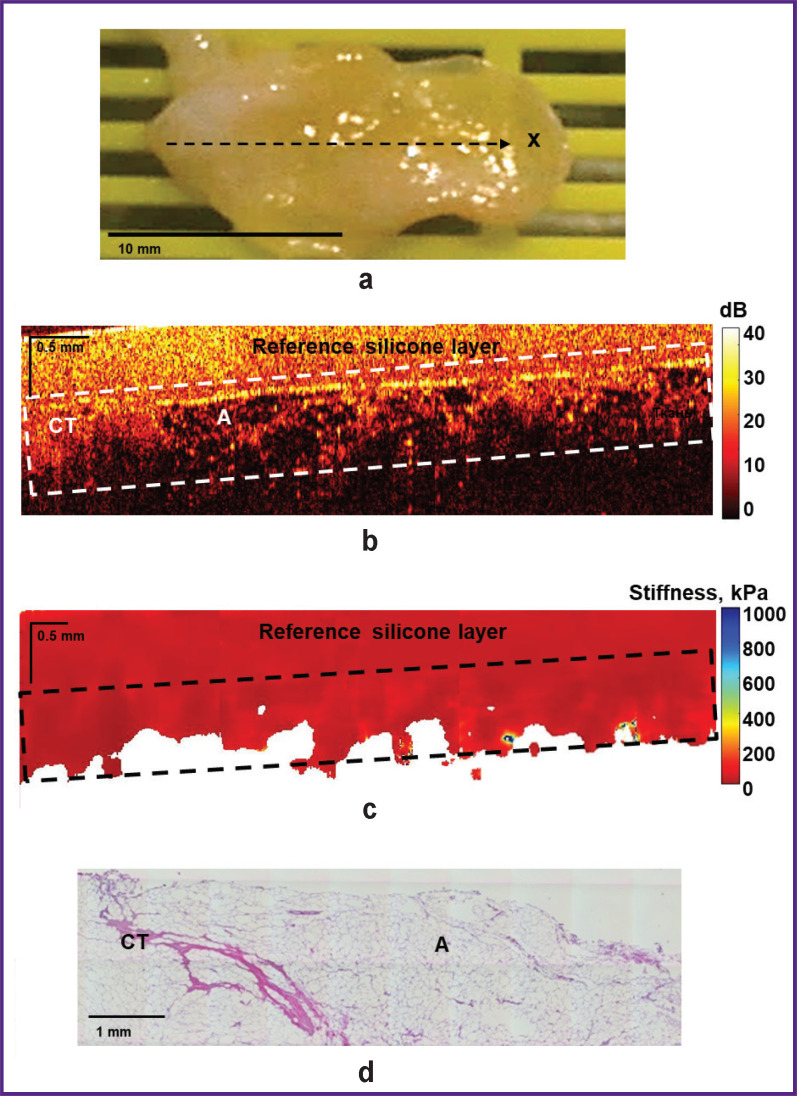
“Negative” surgical margin at the tumor bordary. Adipose tissue with streaks of connective tissue is visualized: (a) a photo of the excised breast tissue sample; (b) a structural OCT image and (c) a corresponding OCE image (obtained by bonding several sequentially scanned sections), where the dotted rectangle marks the area of breast tissue with a homogeneous calibration silicone layer on the surface; (d) histological image stained with hematoxylin and eosin. Here: CT — connective tissue; A — adipose tissue as “negative” surgical margins. They included adipose tissue, adipose tissue with streaks of connective tissue; fibrous tissue with dilated ducts; sclerosing fibrocystic mastopathy or diffuse fibrosis with the lobules and ducts with cystic expansion seen in the margins. In 4 of 100 surgical margin samples examined, the following “positive” surgical margins were found in line with histological reports: invasive BC or carcinoma *in situ*.

“Negative” surgical margins, as it was already mentioned, can include various types of benign tissue. Thus, Figure 3 shows an example of visualization of the “negative” surgical margin, represented by the growth of adipose tissue of the mammary gland (lipomatosis) with streaks of connective tissue, which is characterized by a “cellular” structure on structural OCT images (Figure 3 (b)) with a low level of OCT signal in the adipose tissue and a high level of OCT signal in the connective tissue. The corresponding OCE images (Figure 3 (c)) of intact connective tissue and adipose tissue are characterized by the lowest stiffness values (below 100 kPa).

**Figure 4.**
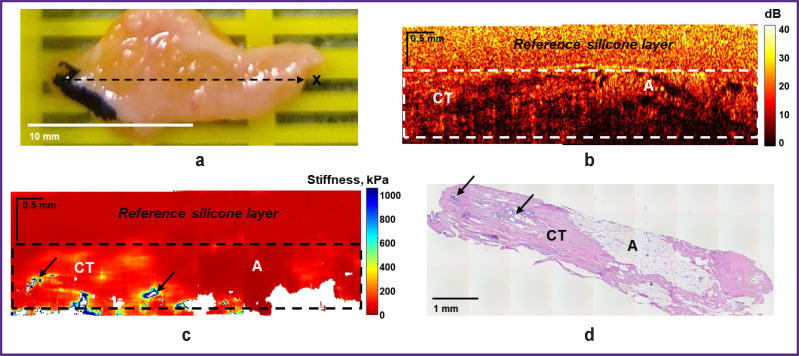
“Negative” surgical margin at the breast tumor margin. Sclerosing fibrocystic mastopathy is visualized with lobules and ducts with cystic expansion (*arrows*): (a) a photo of the excised breast tissue sample; (b) a structural OCT image and (c) a corresponding OCE image (obtained by bonding several sequentially scanned sections), where the dotted rectangle marks the area of breast tissue with a homogeneous reference silicone layer on the surface; (d) a histological image stained with hematoxylin and eosin. Here: CT — connective tissue; A — adipose tissue

Figure 4 demonstrates another example of visualization of the “negative” surgical margin with areas of adipose tissue and larger areas of benign connective tissue (sclerosing fibrocystic mastopathy), which are characterized by a high level of OCT signal on OCT images (Figure 4 (b)) and great penetration depth. The corresponding OCE images (Figure 4 (c)) show both low values of stiffness (below 100 kPa) and slightly increased stiffness in the benign fibrous stroma (100– 200 kPa). In addition, this case demonstrates several points with high values of stiffness (over 400 kPa) (see Figure 4 (c), *arrows*), corresponding to lobules and ducts with cystic dilatation. However, such small (isolated) areas of high stiffness cannot be suspect of tumor cells.

**Figure 5.**
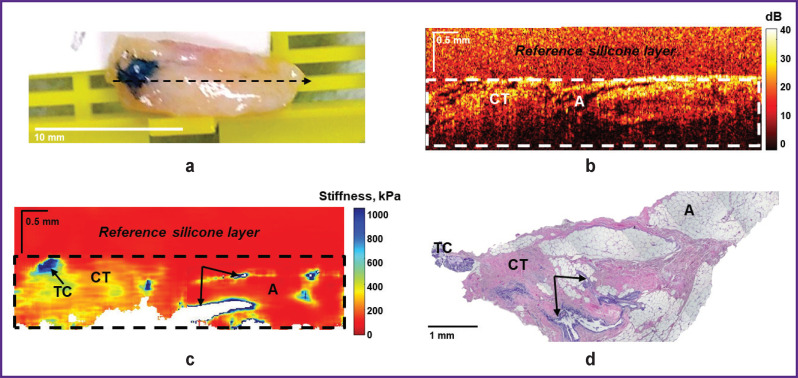
“Positive” surgical margin at the breast tumor margin. Sclerosing fibrocystic mastopathy, dilated intralobular ducts with atypical intralobular hyperplasia and a small tumor cell clusters at the margin are visualized: (a) a photo of the excised breast tissue sample; (b) a structural OCT image and (c) a corresponding OCE image (obtained by bonding several sequentially scanned sections), where the dotted rectangle marks the area of breast tissue with a homogeneous reference silicone layer on the surface; (d) a histological image stained with hematoxylin and eosin. Here: CT — connective tissue; A — adipose tissue; TC — tumor cells

**Figure 6.**
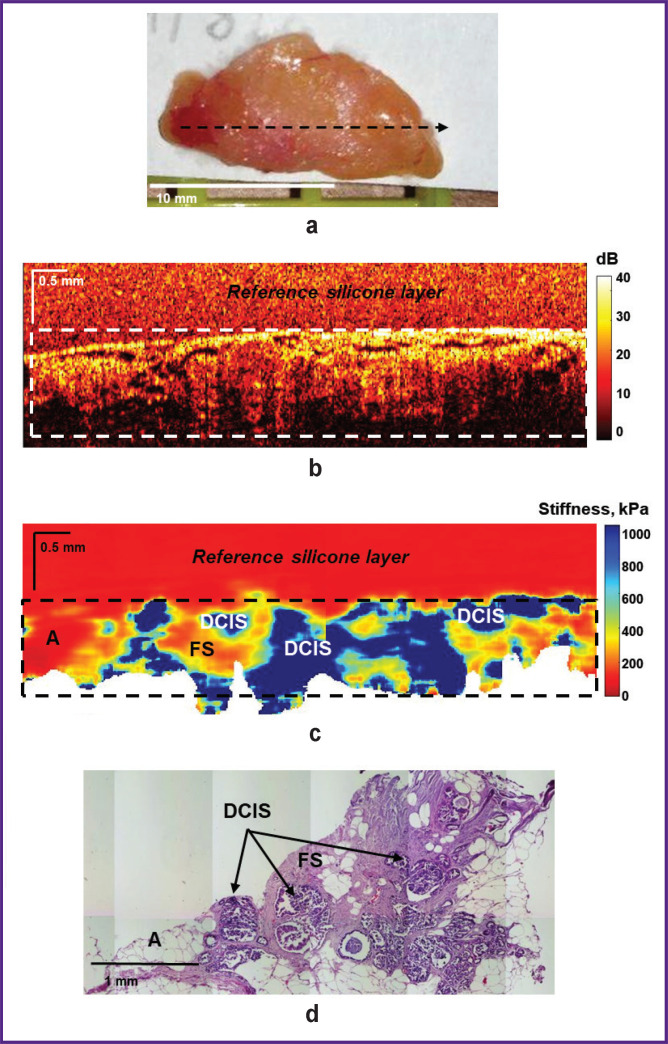
“Positive” surgical margin at the breast tumor margin. Ductal carcinoma *in situ* is visualized with neighboring fibrous stroma and adipose tissue: (a) a photo of the excised breast tissue sample; (b) a structural OCT image and (c) a corresponding OCE image (obtained by bonding several sequentially scanned sections), where the dotted rectangle marks the area of breast tissue with a homogeneous reference silicone layer on the surface; (d) a histological image stained with hematoxylin and eosin. Here: FS — fibrous stroma; A — adipose tissue; DCIS — ductal carcinoma *in situ*

Figures 5 and 6 show examples of a “positive” surgical margin — invasive BC and cancer *in situ*. Figure 5 demonstrates identification of small tumor cell clusters in neighboring non-tumor fibrous tissue with dilated intralobular ducts. Detection of high (over 400 kPa) values of Young’s modulus in the OCE image (Figure 5 (c)) suggests a “positive” surgical margin. Postoperative histological analysis confirmed availability of small tumor cell clusters (over 0.5 mm in size) at the left margin in the neighboring adipose and fibrous tissue (Figure 5 (d)). At the same time, the corresponding OCT image (Figure 5 (b)), in this case, failed to allow identification of tumor cell clusters areas probably due to OCT inability to detect small infiltrations of tumor cells in the neighboring fibrous tissue with a high level of OCT signal.

Figure 6 shows an example of ductal carcinoma *in situ* (DCIS) identification in neighboring fibrous and adipose tissues. As in the previous case, the OCE images compared to the structural OCT images provided more contrasted and precise detection of tumor cells in the surgical margin. Ducts filled with tumor cells in DCIS are visualized as high-contrast areas with highly increased stiffness (over 500 kPa) on OCE images (Figure 6 (c)), which correspond with the histological image (Figure 6 (d)). The neighboring fibrous tissue is characterized by the stiffness values of ~200 kPa, whereas the values of adipose tissue are below 100 kPa.

### Detection of the breast tumor margins in breast-conserving surgery

**Figure 7.**
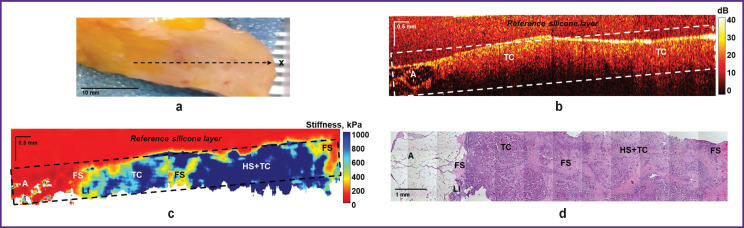
Infiltrating ductal carcinoma of a low degree of malignancy with a transition to non-tumor breast tissue: (a) a clinical photo of a breast cancer sample; (b) a structural OCT image and (c) a corresponding OCE image (obtained by bonding several sequentially scanned sections), where the dotted rectangle marks the area of breast tissue with a homogeneous reference silicone layer on the surface; (d) a histological image stained with hematoxylin and eosin. Here: A — adipose tissue; FS — fibrous stroma; HS — hyalinized stroma; TC — tumor cell clusters; LI — lymphohistiocytic infiltration

Figure 7 shows the results of OCT, OCE, and histological examination of low-grade IDC. OCT and OCE images were obtained throughout the entire tumor node covering also the non-tumor breast tissue. It was found that OCT images of the central region of the tumor node are characterized by a homogeneous and low attenuation rate of OCT signal with depth (Figure 7 (b)). The OCT image shows the margin of the tumor transition into the neighboring adipose tissue, which is identified by its characteristic “cellular” structure with a low level of the OCT signal.

The corresponding OCE images of BC showed an inhomogeneous distribution of high (over 400 kPa) and low (below 400 kPa) Young’s modulus values (Figure 7 (c)), which proved availability of both tumor cells and tumor stroma. At the same time, it should be noted that OCE images visualize the margin of the tumor transition into the breast adipose tissue more clearly and with a better contrast compared to structural OCT images. Based on morphological analysis, the following diagnosis was established: IDC of a solid-scirrhous structure with areas of fibrous stroma and hyalinosis of single collagen fibers, as well as moderately pronounced lymphohistiocytic infiltration, mostly along the tumor margin (Figure 7 (d)).

**Figure 8.**
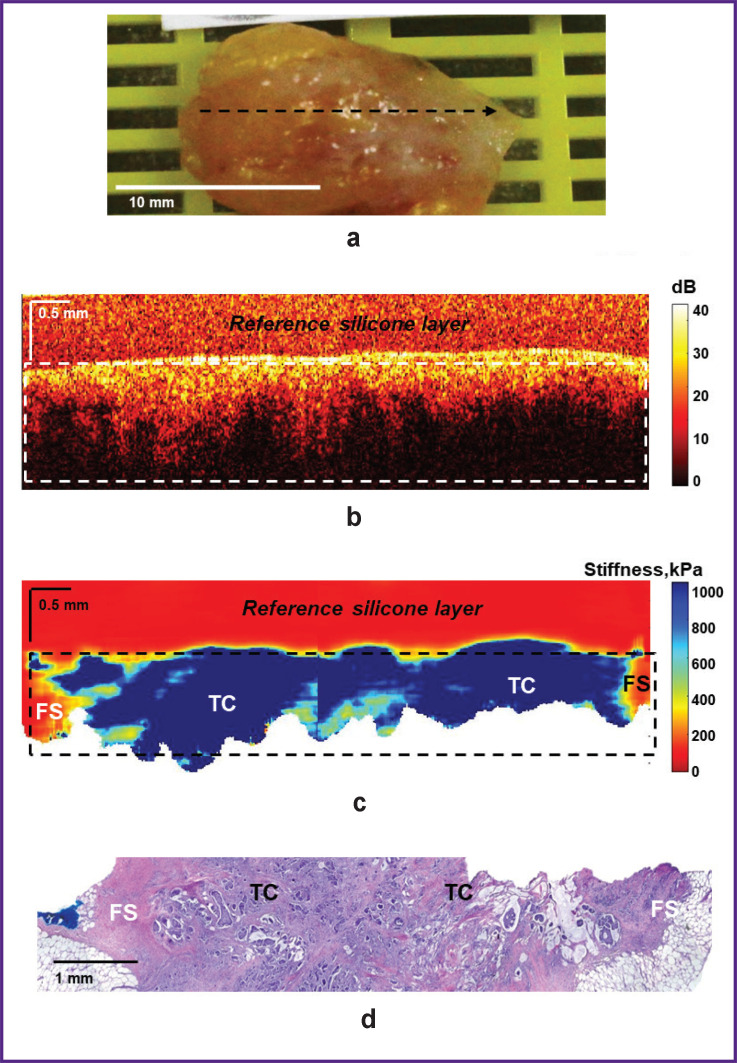
Infiltrating ductal carcinoma of a high degree of malignancy with a transition to non-tumor breast tissue: (a) a clinical photo of a breast cancer sample; (b) a structural OCT image and (c) a corresponding OCE image (obtained by bonding several sequentially scanned sections), where the dotted rectangle marks the area of breast tissue with a homogeneous reference silicone layer on the surface; (d) a histological image stained with hematoxylin and eosin. Here: FS — fibrous stroma; TC — tumor cell clusters

Figure 8 shows the results of OCT, OCE, and histological examination of high-grade ILC. As in the previous case, OCT and OCE images were obtained throughout the tumor node with a transition to the non-tumor breast tissue. These images in the area of the tumor node visualize a homogeneous distribution of a high backscatter level and predominance of high stiffness values (Figure 8 (b) and (c), respectively). At the same time, it is difficult to determine the margin of tumor and non-tumor breast tissues on the OCT image due to the similar level of backscattering of tumor cells and connective tissue at the breast tumor margin. On the contrary, the OCE image shows the tumor clearly and with contrast, it demonstrates high stiffness values in the area of the tumor node (over 500 kPa) and low stiffness values in the neighboring non-tumor fibrous stroma (below 200 kPa). Histological image on Figure 8 (d) shows that it is a solid ILC surrounded by connective tissue with fibrosis and hyalinosis of collagen fibers.

Thus, compared to structural OCT images, OCE images demonstrated higher contrast between breast tissue types and a more precise boundary of tumor tissue to non-tumor tissue transition for various BC subtypes. The results obtained can be used as an accurate intraoperative detection of the surgical margin in BCS.

### Algorithm of MM OCT examination in breast-conserving surgery

**Figure 9.**
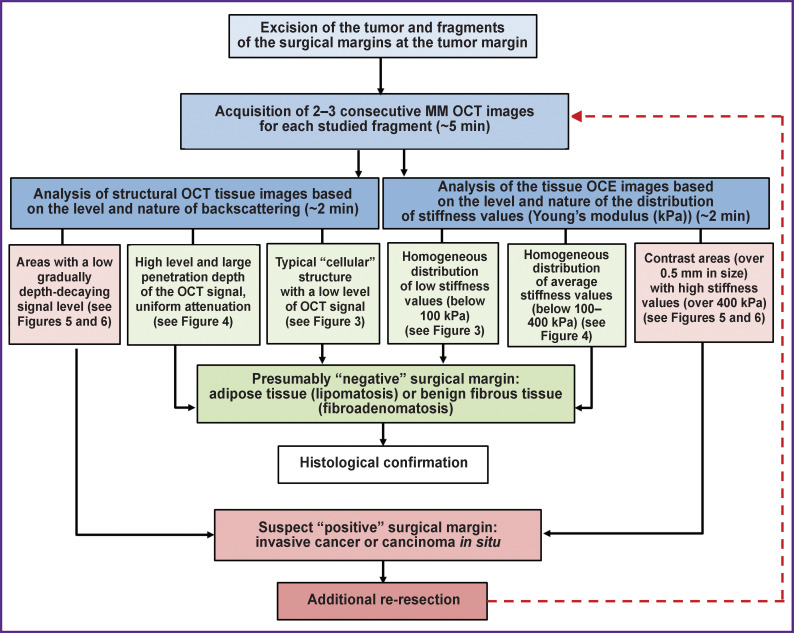
Algorithm for a multimodal OCT study to determine the precise breast tumor margin and assess the surgical margin status in breast-conserving surgery

The conducted study allowed us to develop an intraoperative MM OCT examination algorithm for detecting the exact breast tumor margin and assessing the surgical margin status (Figure 9). The specified time approximately characterizes the duration of the examination of all fragments of tumor and non-tumor breast tissue under analysis.

## Discussion

This study demonstrates high potential of using intraoperative MM OCT examination (with a complex analysis of OCT and OCE images) of tumor margins for various BC subtypes and surgical margin status from several sides of the tumor (according to standard clinical guidelines) in BCS using the developed algorithm for such examination. Compared to histological or cytological analysis, which requires time, OCT can be used intraoperatively to assess tumor margins with high resolution, lable-free and in real-time. The results of using OCE in this study show that the distribution of the absolute values of the tissue Young’s modulus is the most accurate and contrasting predictor of malignancy detection in comparison with traditional structural OCT images indicators. This trend was confirmed for various types of BC as well as for assessment of surgical margin status, demonstrating a significant increase of stiffness (Young’s modulus) in the area of tumor cell clusters. BCS control by examining surgical margins for tumor cells using OCE demonstrated correspondence of the results with histology findings, which confirms the potential of this techniques to be used as an additional intraoperative tool for real-time assessment of the surgical margin status.

Compared with the earlier studies of structural OCT images [18, 19, 41, 42], the current results demonstrate high clinical significance of using the OCE technique according to standard clinical guidelines for achieving a “negative” surgical margin in BCS. The difficulty of interpreting structural OCT images is related to ambiguity of identifying individual structures by the level of backscattering, which is compensated by contrast color-coded OCE images. Several studies [22-26] demonstrated that OCE could detect a malignant tumor in the breast tissue and solve the problem of finding a “negative” surgical margin. This study result is consistent with the results of Allen et al. [24], who used wide-field quantitative OCE to detect “positive” surgical margins in specimens after BCS. They showed high technique sensitivity (100%) and specificity (97.7%) for detection of a tumor within 1 mm from the margin. Moreover, it was empirically established that a high-stiff area can only be considered a tumor if its size is at least 75% of a 1 mm ROI. Such a criterion is required to exclude erroneous diagnosis of small areas of stiff non-tumor stroma as a tumor.

When examining resection margins for tumor cells in BC patients using OCT and OCE in this study, both “negative” and “positive” surgical margins were identified at a distance of below 5 mm from the visible tumor margin, which were examined from four sides of the tumor for each patient according to standard clinical guidelines. During the examination of 25 patients with BC, 4 of 100 studied surgical margin samples from different patients were found to have “positive” surgical margins according to histological reports: invasive BC or DCIS (see Figures 5 and 6). 96 samples were identified as “negative” surgical margins: adipose tissue, adipose tissue with streaks of connective tissue; fibrous tissue with dilated ducts; sclerosing fibrocystic mastopathy or diffuse fibrosis with lobules and ducts with cystic expansion (see Figures 3 and 4).

Furthermore, the standardized tissue pressure techniques were applied in analysis of OCE images of breast tissue obtained by compression, which gives an advantage in comparative data analysis by providing independence from the image acquisition by operator and a reliable assessment of the results. The spatial resolution of OCE images obtained with the utilized device is 30– 50 μm, which allows reliable detection of structures up to ~500 μm in size in the transverse direction and detection of small groups of tumor cell clusters at surgical margins. In future studies, the proposed approach could be combined with the calculation of an additional nonlinearity parameter to detect even smaller groups of tumor cells (below 500 μm in diameter) in the neighboring fibrous stroma, which will significantly increase the sensitivity of the OCE technique for detecting tumor cells and ensuring “negative” surgical margins.

The presence of a tumor in the surgical margins usually leads to a high frequency of local recurrences; however, biological characteristics of the tumor also affect the recurrence and long-term results [43]. This study demonstrates the OCE technique capacity not only to accurately detect the tumor boundaries of various BC subtypes, but also to determine the microstructural features of the tumor based on the stiffness values, which allows determining the degree of aggressiveness (malignancy) of the tumor [22]. Tumors with a more uniform distribution of high stiffness values are more aggressive tumors of high-grade malignancy (see Figure 8). Tumors with a heterogeneous distribution of high and low stiffness values are characterized as less aggressive, with a low grade of malignancy (see Figure 7).

A tumor located near adipose tissue is well visualized both on structural OCT images and on OCE images. However, the breast tumor margin with the neighboring fibrous stroma is better visualized giving higher contrast on OCE images. This study, as well as the earlier studies of the authors [22, 23], demonstrates the OCE ability to detect various degenerative changes in the connective tissue of BC stroma (fibrosis, hyalinosis, etc.) to identify lymphohistiocytic inflammation and to distinguish adipose and connective tissues from tumor cell clusters.

Future studies can analyse OCE sensitivity and specificity and estimate the number of avoided surgeries. This is important because high OCE sensitivity is expected to ensure a clean resection margin. High specificity can prevent unnecessary benign tissue removal, but it is of less importance compared to high sensitivity of the technique in the tumor margin assessment.

It should be noted that the use of OCT and OCE for tumor imaging has limitations due to a small depth of penetration of infrared radiation into tissues (1–2 mm), which makes it difficult to view the entire thickness of the tumor node [44]. Some approaches to address this limitation were suggested in the study of postoperative tissue samples, as well as when using endoscopic and catheter guides [45, 46].

Thus, the OCE technique used to examine tissue stiffness (Young’s modulus (kPa)) demonstrates a high potential for assessing the surgical margin status in BCS. In future, this may provide a reliable local intraoperative control of the surgery efficacy and lead to a significant increase in the relapse-free survival of patients with BC. MM OCT can be used both intraoperatively for surgical resection control and at the preoperative stage for real-time biopsy control.

## Conclusion

The study demonstarets the potential of multimodal (multifunctional) OCT, particularly its OCE mode, as a real-time method for intraoperative tumor margin and surgical margin assessment in BCS. OCT-elastography images compared to conventional OCT imaging, allows to obtain images with higher contrast between different types of breast tissue, (adipose tissue, fibrous stroma, hyalinized stroma, tumor cell clusters), as well as more accurate identification of the tumor border and detection of small groups of tumor cells at surgical margins. OCT elastography demonstrates a high potential for intraoperative detection of a “positive” surgical resection margin at a distance of below 5 mm from the visible breast tumor margin based on detection of tumor cell clusters of approximately 500 μm (0.5 mm) in size and with stiffness of over 400 kPa. The use of MM OCT with an optimal ratio of imaging time, field of view, and resolution makes this technique promising for improving the intraoperative assessment of the surgical margin status and, thus, assessing the adequacy of BCS with the potential of reducing the risk of tumor recurrence. An algorithm for intraoperative MM OCT examination of the state of the resection margin is proposed in accordance with standard clinical guidelines for achieving clean surgical margins in breast cancer patients.
